# Direct Fixation of Acute Chondral-Only Fragments in Young Patients

**DOI:** 10.1177/19476035251351781

**Published:** 2025-07-01

**Authors:** Paul B. Walker, Guillermo Araujo, Mathangi Sridharan, Eghosa Edogun, William L. Sheppard, Thomas J. Kremen, Peter D. Fabricant, Kristofer J. Jones

**Affiliations:** 1 University of California, Los Angeles, Los Angeles, CA, USA; 2 Hospital for Special Surgery, New York, NY, USA

**Keywords:** articular cartilage, general, clinical research knee, clinical research, chondroblasts, patellofemoral studies, clinical research chondrocytes or chondrogeneic stem cells implantation

## Abstract

**Introduction:**

Cartilage lesions of the knee frequently result from acute traumatic injuries and pose significant challenges, particularly in young and active patients. While many involve osteochondral lesions, isolated chondral defects also occur. Traditional treatment focuses on fixation when viable subchondral bone is present; however, managing chondral-only lesions remains controversial due to limited intrinsic healing capacity.

**Methods:**

A systematic review was conducted, screening over 300 studies since August 2023. Inclusion criteria required (1) English-language studies, (2) reports on isolated chondral fragment fixation, and (3) a minimum of 6 months of follow-up. Eighteen studies met these criteria. Data on patient demographics, lesion characteristics, fixation methods, clinical outcomes, and functional scores were extracted.

**Results:**

A total of 76 patients (mean age: 14.3 ± 3.7 years) were analyzed. Males comprised 80.3% of the cohort. The mean follow-up was 40.3 months (range: 7-171), and the mean chondral fragment size was 4.28 cm^2^. The most common lesion locations were the lateral femoral condyle (34.2%), trochlea (32.2%), patella (25%), and medial femoral condyle (8.6%). Healing occurred in 96% of cases, and 86% of patients returned to sports at an average of 10.3 ± 6.1 months. Younger patients (≤14 years) had a significantly higher RTS rate (OR: 5.8; *P* = 0.0427). Functional scores (IKDC, KOOS, Marx, Tegner) demonstrated excellent postoperative outcomes.

**Conclusion:**

Despite prior concerns regarding chondral-only fixation, this study demonstrates high healing rates and favorable functional outcomes. Direct fixation is a viable strategy, particularly in adolescents and young adults. Further prospective trials are needed to validate these findings.

## Introduction

The principal function of articular cartilage is to provide a smooth, lubricated surface for low-friction articulation and to distribute loads to the underlying subchondral bone.^1^ Cartilage lacks blood vessels, nerves, and lymphatics, traditionally showing limited capacity for intrinsic healing and repair. Damage to articular cartilage can lead to significant joint degeneration, manifesting symptoms including pain, swelling, and decreased range of motion.^1,2^

Traumatic cartilage lesions pose significant clinical challenges, particularly in young active patients with large chondral defects. Many of these injuries are initially misdiagnosed as soft tissue injuries, potentially affecting healing potential and leading to poor long-term outcomes.^3^ A large proportion of these injuries involve traumatic osteochondral fragments, typically managed with well-established surgical procedures that yield relatively predictable outcomes.^4-7^ Less commonly encountered are isolated full-thickness chondral fractures, particularly observed in skeletally immature patients following traumatic events such as patellar dislocation.^8^ The pediatric population is particularly susceptible to chondral shear fractures, largely due to incomplete development of the calcified cartilage layer.^9^ Unfortunately, non-operative treatment of chondral fragments is often associated with poor clinical outcomes^3^ and can lead to arthralgia, functional impairment, and eventual degenerative osteoarthritis.^10^ Although concerns regarding the intrinsic healing potential of cartilage-only fragments have historically led to skepticism about the efficacy of fixation in the absence of viable subchondral bone,^11,12^ a growing body of evidence demonstrates that acute fixation of chondral fragments can result in successful healing and favorable clinical outcomes.^12,13^

Traditional management of large (approximately >2 cm^2^), acute, displaced chondral fragment injuries has historically involved a staged surgical approach. This typically includes surgical excision, followed by articular cartilage restoration techniques such as marrow stimulation, autologous chondrocyte-based techniques, and osteochondral allograft implantation if symptoms persist.^14^ More recent case series studies suggest that early fixation of these cartilage-only fragments in young patients can achieve comparable healing rates to fixation of osteochondral fractures, in osteochondritis dissecans (OCD) and traumatic lesions.^11-13,15-34^ This observation may be attributed to the presence of microscopic amounts of bone within the injured chondral fragment.^18^ Fixation of these chondral-only fragments offers several potential advantages, including the preservation of the patient’s own hyaline cartilage, avoidance of donor site morbidity associated with autologous repair techniques, and reduced treatment costs compared to more complex and expensive procedures such as cell-based or composite tissue transfers (e.g. osteochondral allograft) procedures.

This review critically examines the existing literature on the primary fixation of acute chondral-only fragments, emphasizing postoperative clinical outcomes, lesion characteristics, fixation methods, healing rates, and return to sports or activity. Our primary hypothesis is that acute chondral-only fragments in adolescent and young adult patients can achieve reliable healing with favorable patient-reported functional outcomes.

## Methods

### Literature Search

PubMed and Cochrane Library databases were utilized in the development of this systematic review. They were searched on August 2023, using the terms “fixation” and “chondral.” Abstracts and article titles were individually reviewed in conjunction with the PRISMA (Preferred Reporting Items for Systematic Reviews and Meta-Analyses) checklist. Literature was selected and analyzed to ensure that it adhered to the following inclusion criteria: (1) Study in the English language; (2) Reports on cartilage-only fragments (defined as the inability to visualize the fragment on injury radiographs or discern bone on the articular portion of the fragment intraoperatively); and (3) Reports minimum 6 months follow-up. Any meta-analyses, systematic reviews, cadaveric, *in vitro*, and animal studies were excluded for the purposes of this study.

**Figure 1.**
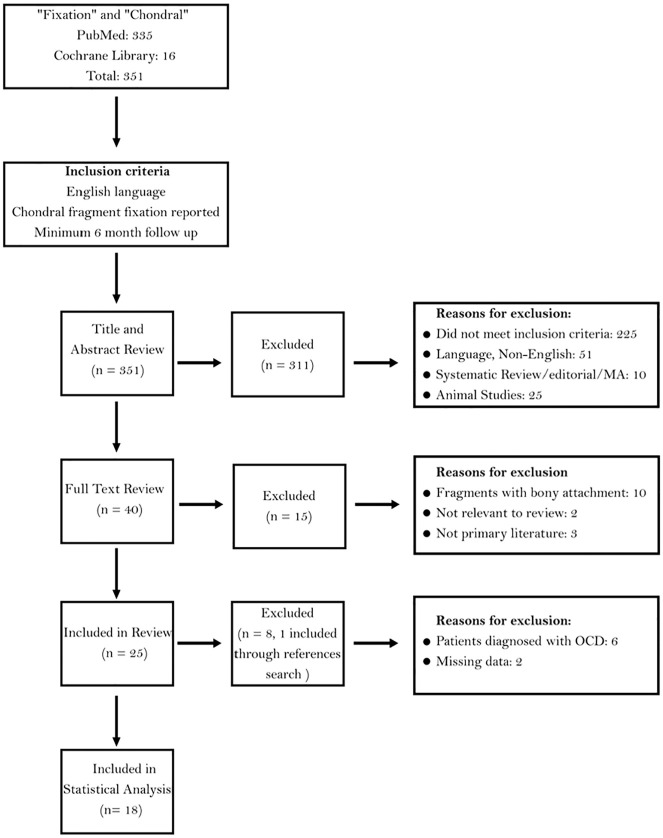
PRISMA (Preferred Reporting Items for Systematic Reviews and Meta-Analyses) flow chart displaying the utilization of inclusion and exclusion criteria.

Our initial search produced 335 articles on PubMed and 16 articles on Cochrane Library, yielding a total of 351 potential articles. Each of these studies was processed and assessed using the Preferred Reporting Items for Systematic Reviews and Meta-Analyses (PRISMA) protocol. Filters were not used in the search to increase the sensitivity of the publications gathered. Furthermore, the title and abstract review of all studies was carried out with predetermined exclusion criteria. This includes the elimination of articles that were published in a language other than English as well as editorial reviews and animal studies. The majority of articles were excluded at this stage due to a lack of relevance to our topic. Full-text reviews were performed on 40 articles, and the reference lists for each article were checked, resulting in the identification of one additional study. Following the application of exclusion criteria, 10 articles that reported on patients with osteochondral fragments were removed, as well as 3 papers that were not primary literature **(Fig. 1)**. Careful attention was taken to ensure that all articles mentioning appreciable amounts of bone on free chondral fragments were excluded from our analysis. A total of 26 papers were then included and underwent thorough review. Six articles reported on patients with primarily OCD-related injuries, and 2 articles were excluded due to missing data,^35,36^ thus not included in our final analysis. Individual patients that did not meet the above-mentioned criteria were carefully removed from the data analysis. A total of 18 articles were included in our final synthesis.

### Data Abstraction

A full-text review of each article took place after inclusion/exclusion criteria were applied. Data on patient age, sex, lesion location, lesion size, fixation method, clinical follow-up, time to surgery, mechanism of injury, and healing rates were abstracted and reported.

## Results

### Demographics

All studies included in our analysis were case series studies or case reports series (level 4 evidence). A total of 76 patients were identified in our review, 61 (80.3%) of whom were male. The mean age was 14.4 ± 3.7 years (range, 10-30 years). The mean duration of clinical follow-up was 40.3 months (range, 7-171). The average reported chondral fragment size was 4.28 cm^2^ (range, 0.8-12.3 cm^2^). The most common locations of chondral pathology were the lateral femoral condyle (34.2%), the trochlea (32.2%), the patella (25%), and the medial femoral condyle (8.6%) **(Table 1)**. The mean time to surgery ranged from 2 days to 4.9 months among all included studies. While some studies did not report a precise mechanism of injury for each patient, we identified a total of 23 patients who sustained patellar dislocations, and 8 of them (34.8%) underwent simultaneous medial patellofemoral ligament repair (MPFL).

**Table 1.** Demographics and Lesion Characteristics of Studies Included in the Analysis.

First Author, Year of Publication	*N* (Patients)	Age in Years, Mean (Range)	Male (Percent)	Lesion Size in cm^2^ Mean (Range)	Lesion Location	Mean Time to Surgery (Days)	Associated Patellar Dislocation
Beckert *et al.*^16^	1	11	100%	4.4	LFC	6	0%
Chan *et al.*^17^	1	12	100%	8.8	LFC	1	100%
Churchill *et al.*^18^	10	13.4 (10-17)	100%	3.80 (1.4-6.6)	LFC (2) T(4) P(4)	17.6	44%
Fabricant *et al.*^13^	15	Median 12.7	73%	Median 4.92	T(5) P(6) LFC (4)	11.2	NR
Jeuken *et al.*^20^	3	12.3 (11-14)	100%	4.67 (2-8)	MFC (2) MFC + T(1)	80	0%
Kjennvold *et al.*^21^	10	15 (12-17)	50%	2.97 (1.92-5.98)	P(7) T(2) LFC (1)	26.3	70%
Morris *et al.*^25^	1	14	100%	6.25	LFC	20	0%
Nakamura *et al.*^26^	1	11	100%	NR	LFC	20	0%
Nakayama *et al.*^27^	1	14	100%	3	LFC	42	0%
Ogura *et al.*^28^	5	11.8 (11-14)	60%	4.36 (0.8-9)	T (5)	55.1	NR
Siparsky *et al.*^29^	3	13	100%	NR	LFC (1) MFC (1) P(1)	NR	100%
Uchida *et al.*^30^	3	12.6 (12-13)	100%	7.33 (4-10.5)	LFC (3)	70	0%
Husen *et al.*^31^	16	14.75 (12-16)	75%	3.41 (1-7.5)	LFC (8) T(4) MFC (3) P(1)	19.5	NR
Lawrence *et al.*^32^	1	18	100%	6	T	NR	NR
Maletius *et al.*^22^	2	19.5 (18-21)	100%	7.38 (6, 8.75)	LFC (1) T(1)	NR	100%
Song *et al.*^33^	1	13	100%	2.88	LFC	NR	0%
Noh *et al.*^12^	1	26	100%	2.72	T	7	100%
Frank *et al.*^34^	1	13	100%	12.25	T	27	100%

Confirmation of proper healing was achieved using MRI and second-look arthroscopy. Additionally, 2 studies demonstrated restoration of a normal osteochondral junction upon histologic assessment.^12,26^ Seventy-three (96%) of 76 patients included in the analysis were noted to have survival of the chondral fragment after primary fixation throughout their respective follow-up periods (assessed by MRI (*n* = 45) with or without second-look arthroscopy (*n* = 14) and functionality). Primary fixation was defined as direct reduction of the fragment with internal fixation, excluding the use of cell-based restorative chondral constructs (i.e., MACI).

### Fragment Fixation Technique

A wide array of fixation methods were reported across the included studies, reflecting significant variation in surgical approach. Bioabsorbable implants—such as pins, darts, sutures, anchors, and tacks—were most utilized, with 12 studies utilizing these methods (*n* = 53). Several studies reported favorable outcomes with these materials.^13,16-18,21,22,26,30^ Non-absorbable autologous bone pegs were used in 7 subjects across 3 studies. Non-absorbable compression screws were utilized in 15 subjects, and non-absorbable suture/meniscus arrows in 17 subjects. Primary fragment fixation with internal fixation was achieved in 100% of subjects **(Table 2)**. However, 2 cases of fixation failure were reported,^28,31^ leading to surgical removal of the unhealed fragment.

**Table 2.** Follow-Up Data and Fixation Methods of Studies Included in Analysis.

First Author, Year of Publication	*N* (Patients)	Return to Sport (Months)	Mean Follow-up (Months)	Post-op Confirmation Method (Arthroscopy, MRI/Histology)	Post-op Confirmation Time (Months)	Fixation Methods	Survival Rate (%)
Beckert *et al.*^16^	1	6	31	MRI and Arthroscopy	MRI: 18, Arthroscopy: 21	Bioabsorbable pins	100%
Chan *et al.*^17^	1	NR	NR	MRI and Arthroscopy	MRI and Arthroscopy: 11.5	Bioabsorbable sutures	100%
Churchill *et al.*^18^	10	NR (8 of 10 RTS)	56	MRI	5 (range 3.5-6)	Bioabsorbable chondral darts, compression screws, SmartNails, metallic compression screws	100%
Fabricant *et al.*^13^	15	median: 5.98	median: 12	MRI	median: 12	Bioabsorbable tacks, screws, sutures, sutures plus anchors	93.3%*
Jeuken *et al.*^20^	3	mean: 8	12	MRI	mean: 12	Fibrin glue + suture	100%
Kjennvold *et al.*^21^	10	median: 9	61.2	MRI	range 2-9 years	Bioabsorbable meniscus arrows	100%
Morris *et al.*^25^	1	12	12	MRI	12	Bioabsorbable pins	100%
Nakamura *et al.*^26^	1	6	33	MRI, Arthroscopy, and Histology	MRI: 9, Arthroscopy and Histology: 6	Bioabsorbable pins	100%
Nakayama *et al.*^27^	1	7	26	MRI and Arthroscopy	MRI: 4, Arthroscopy: 12	Bone Pegs	100%
Ogura *et al.*^28^	5	mean: 7 (4 of 5 RTS)	62.4	MRI	3 years (range: 1-5)	Bone Pegs	80%
Siparsky *et al.*^29^	3	mean: 22	16	MRI and Arthroscopy	MRI: 3, 14, Arthroscopy: 8	Chondral darts and biologic adhesive	100%
Uchida *et al.*^30^	3	7-24, 1 patient NR	24	MRI and Arthroscopy	MRI and Arthroscopy: 24	Bioabsorbable pins	100%
Husen *et al.*^31^	16	NR (12 of 16 RTS)	41.06	MRI	mean 15.8	Chondral darts, SmartNails, anchors, compression screws	93.7%
Lawrence *et al.*^32^	1	6	24	Clinical	NR	PushLock Anchor	100%
Maletius *et al.*^22^	2	7.5	7.5	Arthroscopy	3.2 and 3.7	Bioabsorbable pins	100%
Song *et al.*^33^	1	24	24	Arthroscopy	18	Bone pegs	100%
Noh *et al.*^12^	1	NR	NR	MRI, Arthroscopy, and Histology	MRI: 21, Arthroscopy and Histology: 21	Prolene Suture	100%
Frank *et al.*^34^	1	10	10	Arthroscopy	2.3	Chondral darts	100%

* One patient experienced a fall 8 weeks postoperatively, necessitating reoperation for fragment excision.

### Return to Sport

RTS data were reported in 12 studies included in our analysis. Out of 58 patients, 50 (86.4%) were able to return to athletic activity, with a mean return time of 10.3 ± 6.1 months (6-24 months) and a mean age of 14.44 ± 3.8 years. Notably, while 8 patients (mean age of 15.88 ± 4.22) did not return to sports, 6 (66.7%) of them demonstrated healed fragments confirmed via second-look arthroscopy or MRI. Although the percentage of males among patients who returned to sports was lower compared to females (85.1% vs. 90.9%), there were no statistically significant differences between the sexes (OR: 0.57, [95% CI, 0.06-5.19]; *P* = 0.62). The majority of our study participants were early adolescents (*n* = 52, 68%). This cohort exhibited a notably increased likelihood of RTS compared to individuals aged 15 years or older (94% vs. 74%, OR: 5.8 [95% CI, 1.06-32.00]; *P* = 0.0427).

### Functional Outcomes Scores

Four studies included in the analysis reported functional outcomes.^18,21,28,31^ **Table 3** summarizes the reported outcomes, and the average reported scores. Regardless of the fixation method, nearly all patients returned to activities of daily life and sports.

**Table 3.** Functional and Patient-Reported Outcome Scores (IKDC, KOOS, Marx, Tegner, and PROMIS).

						Single-Hop Test		PROMIS	
	IKDC	KOOS	Marx Activity Scale	Tegner	Lyshom	Uninjured	Injured	Physical %	Physiol.% %
Mean post-op	95.1	96.6	14	6.2	92.5	141	127	93.8	90.6

### Post-Operative Protocol

Regrettably, the larger studies^13,18,31^ failed to provide detailed information on postoperative protocols,^13,18,31^ as these were largely dependent on the surgeon’s preferences. Conversely, smaller studies tended to indicate brace or cast immobilization at 0°, 30°, or 45° for a period of 3 to 6 weeks,^12,21,22,26,27,29,30,33,34^ or immediate knee flexion progression following surgery.^16,17,20,25,28^ Typically, patients were prohibited from weight-bearing after the surgical procedure, with partial weight-bearing allowed at 5 to 8 weeks and full weight-bearing at 8 to 12 weeks in the postoperative phase.^16,17,26,27,29,32,33^

## Discussion

This meta-analysis provides strong evidence that direct fixation of isolated chondral-only fragments in young patients is a safe, effective, and a biologically viable treatment strategy with consistently favorable outcomes. Across 18 studies comprising 76 patients, fragment healing was achieved in 96% of cases, as confirmed by postoperative MRI and/or second-look arthroscopy. Functional outcome scores, where available, were uniformly positive, reflecting restoration of joint function and return to pre-injury activity levels. Return-to-sport data, reported in 12 studies, demonstrated that 86.4% of patients resumed athletic participation at a mean of 10.3 months following surgery. Notably, patients aged 14 years or younger exhibited significantly higher RTS rates than older adolescents, reinforcing that chondral-only fixation may have greater reparative potential in early adolescence. These findings challenge the historical belief that cartilage-only lesions lack intrinsic healing capacity and support the growing role of primary fixation in the management of acute chondral injuries. While various fixation techniques—including bioabsorbable implants, autologous bone pegs, and compression screws—were employed, the uniformly high success rates across methods indicate that outcomes may be more influenced by patient age, fragment viability, and timely intervention than a specific fixation construct.

The complexity of this topic is underscored by the rarity of cartilage-only lesions and the historical belief that cartilage defects, when isolated, cannot heal to bone.^22,24,25^ Notably, one study, which included patients from 2 outpatient clinics between January 2000 and December 2009, reported an incidence of 3 out of 6,000 patients presenting with an isolated chondral fragment.^30^ As illustrated in **Table 1**, adolescent patients represent the majority of those at risk for pure chondral injuries, as their incomplete formation of a calcified cartilage layer results in a relatively weak tissue interface between the articular cartilage and the underlying subchondral bone.^9,37^

A wide array of fixation techniques has been described for the treatment of isolated chondral fragments. These include bioabsorbable constructs—such as sutures, tacks, screws, anchors, pins, and darts—as well as non-absorbable options like metallic compression screws, autologous bone pegs, and meniscus arrows. Among these, bioabsorbable implants were the most frequently utilized in the reviewed studies, likely due to their ability to eliminate the need for secondary hardware removal. Favorable clinical outcomes have been reported with bioabsorbable fixation techniques^13,16-18,21,22,26,30^; however, complications such as implant-mediated synovitis have been observed, particularly with poly-L-lactic acid (PLLA)-based materials, which are commonly used in biodegradable devices. Innovative approaches, including fibrin glue augmentation^20^ and Prolene suture constructs,^12^ have also been described, although the available data remain limited. While no single fixation method was found to be superior, likely due to the underpowered nature of the included studies, healing rates were consistently high, with 96% of patients (73 out of 76) demonstrating successful fragment healing as assessed by MRI or second-look arthroscopy.

Functional outcome scores among the patients included in this study supported these findings, with consistently excellent postoperative results in 4 studies.^18,21,28,31^ There were 2 instances of fixation failures observed,^28,31^ with Ogura *et al.*^28^ attributing the failure specifically to a large chondral fragment size (9 cm^2^). The high success rate of primary fixation in treating isolated chondral fragments may be partly attributed to the presence of microscopic bone tissue at the osteochondral interface. This characteristic may facilitate healing processes similar to osteochondral allograft incorporation, as demonstrated in a bovine *in vitro* model. In this model, adult cartilage delaminates within a well-defined region, while immature tissue separates into the subchondral bone, resulting in deeper penetration of cartilage tissue and a less distinct osteochondral transition zone.^38^ Collectively, these basic science and clinical studies suggest that repairing isolated chondral fragments is a viable treatment option, particularly in young patients and in acute settings.

The average return to sport (RTS) across studies was 10.2 months, with an overall RTS rate of 86.4%, and no differences observed between sexes. However, age and injury characteristics appear to influence RTS outcomes. Fabricant *et al.*^13^ reported a 100% RTS rate at a median of 26 weeks among 15 skeletally immature patients treated at 2 tertiary children’s hospitals, all of whom underwent fixation with bioabsorbable implants. In contrast, Husen *et al.*^31^ reported a lower RTS rate of 73% in a cohort of 16 patients treated with chondral darts and compression screws; notably, 4 of the 5 patients who failed to return were over 14 years old. Similarly, Churchill *et al.*^18^ reported failure of RTS in 2 of 10 patients (aged 17 and 25 years) using comparable fixation techniques. These findings suggest a potential age-related trend favoring earlier return in younger patients.

The observed age-related differences in RTS outcomes may be partially explained by biological differences in cartilage healing potential.^5^ Skeletally immature individuals have been shown to possess enhanced reparative capacity, possibly due to a greater population of resident chondroprogenitor cells within the cartilage layer.^39^ This is supported by findings from animal models, which demonstrate superior healing responses in immature cartilage compared to mature tissue.^40,41^ In our analysis, early adolescents had a 5.8-fold higher likelihood of returning to sport compared to older patients (95% CI, 1.06-32.00; *P* = 0.0427). However, the ability to definitively stratify patients by skeletal maturity was limited due to incomplete or inconsistent reporting across studies, as well as the relatively small sample size.

Beyond age and fixation technique, RTS timing and variability were likely influenced by the nature of the initial injury. Given the high frequency of lesions involving the patella, lateral femoral condyle, and trochlea—regions commonly injured during patellar dislocation—it is probable that many patients sustained a dislocation event, even when not explicitly reported. Since healing of the chondral fragment was consistently achieved across fixation types, the initial traumatic mechanism, such as patellar dislocation, may have had a more significant impact on RTS outcomes.

The most common concurrent procedure reported was medial patellofemoral ligament (MPFL) reconstruction, typically performed in the setting of patellar instability. Although only 23 dislocations were explicitly reported, lesion locations suggest that most injuries occurred in this context. MPFL reconstruction is a well-established adjunct in adolescent patients at risk of recurrent instability and has been shown to significantly reduce the likelihood of redislocation.^42,43^ Several studies included in our review reported subsequent surgeries for patellar stabilization within 1 to 2 years of the index procedure.^13,17,21^ Therefore, whether or not patients underwent MPFL reconstruction may also have influenced RTS outcomes.

Unfortunately, the larger studies^13,18,31^ did not provide comprehensive details on postoperative protocols, as these were predominantly influenced by surgeon preferences. In contrast, the studies that did outline postoperative protocols exhibited substantial variation. These differences included the use of cast or brace immobilization at specific knee angles versus immediate knee flexion progression after surgery. Additionally, some studies recommended immediate toe-touch weight bearing, while others advised against weight bearing for varying durations. These discrepancies can be attributed to the diversity of intraoperative findings, particularly regarding whether the size of the lesion, whether it affected a weight bearing surface, and the presence of concomitant procedures for patellar instability.

There are several limitations to this study, including inconsistent reporting of patient demographics, surgical details, and clinical outcomes. While overall survival and RTS rates are reported to be high across all studies, there is a critical need for consistent collection of modern patient-reported outcome measures to assess the effectiveness of each treatment strategy more accurately. Functional outcome scores were fully documented in only 4 out of 18 studies; the remaining studies lacked comprehensive patient-reported outcome measures. Moreover, all included studies were classified as level 4 evidence, with no randomized controlled trials or control groups available for comparison. Additionally, a minimum clinical follow-up of 6 months was utilized to ensure an adequate sample size, which may potentially overestimate the healing rates and long-term functional outcomes. While short-term fragment healing was consistently observed, the limited duration of follow-up precludes assessment of long-term chondral durability. It is a common clinical observation that many of these fragments exhibit surface fissuring, and although early incorporation is often successful, the damaged cartilage surface may predispose the fragment to earlier degeneration compared to surrounding healthy cartilage. Longer-term follow-up will be necessary to fully evaluate the durability and longevity of fixation in this population.

In conclusion, this systematic review and meta-analysis included 18 studies involving 76 subjects under the age of 30, all with chondral-only defects primarily treated with direct reduction and internal fixation. While methods of fixation varied among studies, functional outcomes consistently improved postoperatively, with a high survival rate of 96% assessed predominately by MRI and second-look arthroscopy. The average RTS rate was 86% at 10.3 ± 6.1 months. Notably, early adolescents demonstrated a higher RTS rate of 94.3%. Our findings support the efficacy of direct fixation for chondral-only lesions. However, additional prospective trials are necessary to validate and further evaluate these outcomes.
